# How the COVID-19 Pandemic Affected Young People’s Mental Health and Wellbeing in the UK: A Qualitative Study

**DOI:** 10.1177/07435584231151902

**Published:** 2023-02-02

**Authors:** Samantha Pearcey, Lowrie Burgess, Adrienne Shum, Eshal Sajid, Milly Sargent, Marie-Louise Klampe, Peter J. Lawrence, Polly Waite

**Affiliations:** 1 University of Oxford, UK; 2 Patient and Public Involvement and Engagement Representative, London, UK; 3 Patient and Public Involvement and Engagement Representative, Oxfordshire, UK; 4 University of Southampton, UK; 5 University of Reading, UK

**Keywords:** child, adolescent, young people, mental health, pandemic, COVID-19, qualitative

## Abstract

There is emerging evidence of the detrimental impact of the pandemic and associated restrictions on young people’s mental health in the UK, but to date, these data have been largely quantitative. The aim of the current study was to gain a deeper understanding of young people’s experiences in relation to their mental health and wellbeing during the pandemic. Seventeen young people, aged 11 to 16 years, sampled for diverse characteristics, and living in the UK, were interviewed virtually between December 2020 and February 2021. Reflexive thematic analysis was carried out by the research team, which included two young people, and five themes were developed: (1) positives; (2) worries and anxiety; (3) sadness and anger about losses; (4) mental exhaustion; and (5) support from others. Aspects of young people’s individual circumstances (e.g., pre-existing mental health difficulties, special educational needs, and neurodevelopmental disorders) appeared to play a role in their experiences. Continued measurement of young people’s mental health, initiatives to identify young people who have been struggling, and the provision of support (including evidence-based and accessible interventions) will be important for protecting young people from future adversities as we emerge from the pandemic.

## Introduction

The restrictions put in place to mitigate the impact of COVID-19 have caused significant disruption to the lives of young people, despite the lower risk of severe health consequences resulting from COVID-19 for young people, relative to other groups. There is emerging evidence of the detrimental impact of the pandemic and these restrictions on young people’s mental health in the UK (Creswell et al., 2021). However, to date, these data have been largely quantitative in nature. Taking a qualitative approach enables a more in-depth exploration of young people’s experiences. This allows us to further understand how the pandemic has affected young people’s mental health and the factors which affect their coping. This is important to inform future decisions about the best ways to support young people in the years following the current pandemic and in future public health crises.

Reports suggest that in broad terms, the COVID-19 pandemic has had an adverse effect on the mental health of children and young people in the UK. Data from the NHS Digital Survey of children and young people’s mental health in England in July 2020 showed a 16% increase in probable mental health disorders compared to 2017 (NHS Digital, 2020). The COVID-19: Supporting Parents, Adolescents and Children in Epidemics (Co-SPACE) study carried out monthly surveys of children and young people’s mental health over the course of a year from March 2020. It found increases in parent/carer-reported symptoms of behavioral and attentional difficulties in children and young people at times of national lockdown and peak restrictions. Greater changes were observed in the younger age group (4–10 years) than the adolescent group (11–16 years) (Creswell et al., 2021). Raw et al. (2021) showed that over the first national UK lockdown (April–May 2020), those with elevated symptoms were more likely to be from a lower income household, have a parent or carer with higher levels of psychological distress, have special educational needs and/or a neurodevelopmental disorder, and to be younger in age.

Nonetheless, there is evidence that many young people did not experience high levels of distress and that some groups experienced improvements in their mental health during the pandemic. For example, over the course of the first national UK lockdown, Raw et al. (2021) found that 49% to 68% of the children and young people in the sample were reported to have “stable low” levels of emotional, behavioral, and attention/hyperactivity difficulties—although it is important to recognize that the sample was not nationally representative. Similarly, Widnall et al. (2020) found that most students in their study adapted well to the school closures during the first national lockdown, and showed a decrease in anxiety compared to before the pandemic.

This variability in experience and coping can be understood in terms of risk and resilience factors. Young people’s ability to adapt in adverse circumstances appears to relate to a range of factors associated with the child or young person, their families, and characteristics of their wider social environments (Luthar et al., 2000). These factors can act as “developmental assets” in that they broadly promote positive outcomes but can also serve as a buffer for risk and may support resilience in challenging circumstances (Roehlkepartain & Blyth, 2020). For example, close relationships, having a supportive family and an internal locus of control have been shown to contribute to aspects of resilience, such as increased coping skills (Shulman, 1993) and decreased vulnerability to life stress (Weist et al., 1995).

Qualitative research on the experiences of children and adolescents during (typically early stages of) the pandemic in the UK and other countries has highlighted the emotional impact of the pandemic on young people (Burgess et al., 2022; McKinlay et al., 2022; O’Sullivan et al., 2021; Pellicano et al., 2022; Scott et al., 2021). Increased levels of stress and anxiety were commonly described across studies (Burgess et al., 2022; McKinlay et al., 2022; O’Sullivan et al., 2021; Pellicano et al., 2022; Scott et al., 2021); emotional difficulties were described in relation to the disruption to young people’s education (e.g., home-schooling and uncertainty about their educational futures), loss of activities and routine, and worries about COVID-19 more generally (e.g., loss of loved ones and absence of clarity around COVID issues). In two of the studies, participants reported that their mental health had deteriorated during the pandemic (Pellicano et al., 2022; Scott et al., 2021) and support from school and services was difficult to access (Burgess et al., 2022; McKinlay et al., 2022; Pellicano et al., 2022).

Young people interviewed in qualitative studies also commonly discussed changes to social relationships and feelings of loneliness and isolation during the pandemic. Some found it difficult to adapt to and maintain social relationships online (McKinlay et al., 2022; O’Sullivan et al., 2021) and reported increased levels of family conflict and feeling trapped within the home (McKinlay et al., 2022; O’Sullivan et al., 2021; Scott et al., 2021). Of note, this included young people who ordinarily may have difficulties in social relationships and communication; for example, Pellicano et al. (2022) noted that, while autistic young people and their families initially felt relieved by the decrease in social pressure, this was quickly overshadowed by a sense of missing in-person social contact and finding online interactions exhausting. In adolescence, peer relationships are of primary importance as young people move away from a reliance on parents/carers for support and interaction (Brown & Klute, 2006); there is some evidence that as adolescents get older, they experience more negative feelings and loneliness when spending time with parents and less when spending time with peers (Goossens & Marcoen, 1999). Thus, the Covid-19-related restrictions on social contact with peers may have been especially challenging at this stage of development.

Despite this emerging evidence, there are gaps in our understanding of the experiences of young people in the UK. Two studies (O’Sullivan et al., 2021; Pellicano et al., 2022) come from outside the UK, where the course of the pandemic and associated restrictions differed. Where studies have been conducted in the UK, participants have been across broad age ranges or selected on the basis of specific demographic variables. For example, two studies (Burgess et al., 2022; McKinlay et al., 2022) included both adolescents and young adults, where there are likely to be differences in education/employment and living arrangements. Scott et al. (2021) interviewed participants from one geographical region of the UK and the study by Burgess et al. (2022) was focused specifically on racially minoritized young people. Finally, much of the qualitative literature to date has focused on earlier experiences in the pandemic, with only one study covering time points after restrictions were tightened again (e.g., up to January 2021; McKinlay et al., 2022), omitting the specific experiences of both the second UK national lockdown and the prolonged effects of the pandemic over time.

Consequently, the aim of this study was to address these limitations by exploring the experiences of young people aged 11 to 16 years, in relation to their mental health and wellbeing and how they coped during the COVID-19 pandemic in the UK. In particular, it set out to investigate young people’s experiences of national lockdowns (April–June 2020; January–March 2021), with interviews taking place during a unique period of easing and tightening of restrictions (December 2020–February 2021). The study purposively sampled for young people across the UK from a range of backgrounds, including those who may be at increased risk of mental health difficulties, such as those with a pre-existing mental health difficulty, special educational needs, and neurodevelopmental disorders.

The study’s research questions were:

(1) What were the experiences of young people aged 11 to 16 years, in relation to their mental health and wellbeing and how they coped during the COVID-19 pandemic in the UK?
(2) What were the experiences at different time points (e.g., during periods of lockdown) and over time as the pandemic progressed?

## Methods

The present study was part of a wider mixed-methods research study (Co-SPACE), tracking the mental health of children, young people, and their families during the COVID-19 pandemic. Ethical approval for the study was granted by the Oxford Central University Research Ethics Committee (Oxford CUREC; Reference: 69060).

### The Research Team

The research team consisted of researchers with an interest in mental health in children and young people, particularly in relation to understanding what causes and maintains mental health difficulties and the development of psychological interventions. As a group, we were largely psychologists by training. Two members of the team (LB and MK) undertook this research as part of their undergraduate (psychology) degrees. PL and PW were both trained clinical psychologists, as well as being parents. ES and MS were young people aged 15 to 16 years, involved as lived experiences researchers. Both young people were interested in young people’s mental health and potentially pursuing a degree in psychology. They were recruited via the Co-SPACE patient and public involvement and engagement (PPIE) group and were involved in the current study to give better representation and understanding of adolescents’ experiences in the analysis. They were not participants in the study. They were involved in analysis of the data and reviewing the final manuscript to ensure that it accurately reflected young people’s experiences. They were financially reimbursed for their work in line with NIHR agreed payment and reimbursement rates for involvement (NIHR, 2020). Three of the team (SP, PL, and PW) had prior experience of qualitative methodology and team discussions always involved facilitation from those with this prior experience. However, the combination of perspectives and experiences within the group facilitated a rich and open discussion about the data.

### Participants and Sampling

To be included in the study, participants were required to be living in the UK and be a young person aged 11 to 16 years. Participants were selectively sampled based on demographic data provided as part of the Co-SPACE survey or via the expression of interest form for external participants. As is typical in qualitative research, we adopted purposive sampling by selecting participants who would be likely to provide information-rich data to analyze (Braun & Clarke, 2013; Patton, 2014), rather than aiming for a generalizable, representative sample. We sampled for individuals based on location within the UK, gender, ethnicity, household income, presence of special educational needs (SEN), neurodevelopmental disorders (ND) or mental health difficulties, physical health difficulties, or those who were fostered/adopted.

The participants for this study were 17 adolescents aged between 11 and 16 years. Fourteen of the participants were recruited through the Co-SPACE survey, completed by their parent/carer (OSF; https://osf.io/8zx2y/), two through involvement in PPIE activities and one through contact with foster agencies. A summary of participants’ characteristics can be found in Table 1.

**Table 1.** Participants’ characteristics.

Variable	Frequency	Frequency
Gender	Boys/young men	5
	Girls/young women	12
Age (years)	11–13	6
	14–16	11
Location	London/Greater London	3
	Southern England	8
	Northern England	4
	Scotland	1
	Wales	1
	Northern Ireland	0
Household income (p.a.)	<£30,000	5
	>£30,000	9
	Prefer not to say	3
SEN/ND	Yes	5
	No	12
Diagnosed mental health difficulty	Yes	4
	No	13
Physical health difficulty	Yes	2
	No	15
Fostered/adopted	Yes	1
	No	16

*Note.* SEN/ND = special educational needs and/or neurodevelopmental disorder. For adolescents who were recruited through participation in the Co-SPACE survey, this reflects the age provided when the survey was first completed and for those recruited through alternative routes, this was provided by the adolescent at the time of recruitment.

We evaluated the adequacy of the sample size continuously during the research process. Our final sample was determined to have high levels of information power, for example, having strong dialog and a combination of participants who were well-specified to answer the research questions (Malterud et al., 2016). As is typical in qualitative research (Braun & Clarke, 2021), we also made a pragmatic decision around the sample size by ensuring that we completed all interviews before the end of the second national lockdown so that participants were reflecting on experiences over the same period.

### Procedure

We invited parents/carers taking part in the Co-SPACE survey to indicate if their child was interested in participating in this qualitative study, as well as contacting parents/carers and young people through other means (e.g., PPIE activity and other organizations) to complete an expression of interest form. Parents/carers from a first round of recruitment were initially contacted based on specific characteristics for which we were purposively sampling. Following low uptake, however, all who had expressed an interest were then contacted. A second round of recruitment was then conducted, and participants were contacted purposively from those who had expressed an interest. In total, 142 parents/carers indicated that their child would be interested in participating and 35 responded to invites to interview. Of these, 20 completed and returned consent forms. All adolescents whose parent/carer had provided written consent were sent information about the study and invited to take part. Nineteen provided written assent and 17 were interviewed. Those who were not interviewed either did not respond to email invitations for an interview or could not fit the interview into their schedule.

Interviews were conducted between December 2020 and March 2021. During this time, the UK had experienced two national lockdowns (from March 2020 to June 2020, and December 2020 to March 2021). During these lockdowns, schools were closed to most children and young people. While at a national level, schools were open from September to December 2020, high levels of restrictions remained in place (e.g., not being able to socially mix with other households indoors), and local lockdowns were in place in some areas of the UK where infection levels were high. Prior to the interview, participants were sent a visual timeline of pandemic-related lockdowns/restrictions and other key events (specific to the devolved nation in which they were residing) to aid discussion.

The first 10 interviews were conducted by a third-year undergraduate psychology student (LB), and a further seven interviews were conducted by a graduate research assistant (AS). Both interviewers identified as women; LB was from a White ethnic background and AS was from an Asian ethnic background. Interviewers received ongoing training and supervision throughout the interview process from clinical research psychologists (PW and PL) and a post-doctoral researcher (SP), experienced in qualitative methods. Participants had not met the interviewers before the interview.

Interviews were conducted on a video call via Microsoft Teams (*n* = 13), with no video via Microsoft Teams (*n* = 2), or over the phone (*n* = 2). At the beginning of the interview, parents/carers and young people gave verbal consent/assent to participate. The interviewer then explained that the purpose of the study was to supplement knowledge from the wider Co-SPACE study and to learn about people’s experiences in greater depth than the survey allowed (and, for the first 10 participants only, that it would also be used as part of an undergraduate thesis). Subsequently, parents/carers were invited to leave the room but asked to remain nearby in the case of distress; as such, most interviews were conducted with the adolescent alone, except where participants opted to have a parent/carer present (*n* = 2), or if the interview was conducted in an area where others were in the background (*n* = 4).

Interviews began with a broad question around how things had been during the pandemic, before moving on to questions around their experiences at times of high restrictions/lockdown and less restrictions (e.g., schools reopening) and the impact on their wellbeing. A topic guide was used flexibly to provide prompts throughout the subsequent discussion (see Supplemental Materials). At the beginning of the interview, participants were reminded that they did not have to answer questions if they preferred not to and that they could request to stop the interview at any point without having to give a reason. At the end, all participants received information about appropriate sources of support and resources that they could access. If any concerns arose in relation to risk/safeguarding, interviewers were required to discuss this with PW, a qualified clinical psychologist, and local safeguarding procedures were followed.

Interviews lasted between 33 and 57 minutes. Following the interview, participants were emailed a £30 gift voucher to reimburse them for their time. Interviews were recorded and transcribed through Microsoft Teams. Back up recordings were made on an external audio recording device. Transcripts were checked for accuracy, amended, and anonymized by the interviewer, as well as sent to participants for confirmation of accuracy. Where transcripts were returned by participants with edits (*n* = 2), these were used for analysis in place of the original transcripts. Field notes were also kept by all interviewers, reflecting their thoughts and observations following each interview to provide additional context where needed during analysis.

### Data Analysis

Data were managed in Nvivo (for Mac and PC) and analyzed using an inductive approach to reflexive thematic analysis, following Braun and Clarke’s six-phase methodology (Braun & Clarke, 2006), including familiarization, generating codes, searching for themes, reviewing themes, defining and naming themes, and producing the report. Transcripts of the interviews were coded by two of the authors (LB, AS), and codes were reviewed by SP.

Initial themes and subthemes were developed from the initial 10 interviews (LB, AS, SP, MLK, PW) by organizing codes into coherent groups. Following further interviews and resulting new codes, the thematic structure was reviewed and developed iteratively by the study team over several meetings. SP and PW further refined the thematic structure between meetings. The lived experience researchers (MS and ES) were actively involved in research group meetings to develop themes and subthemes. ES and MS had met some members of the research group before the data analysis meetings. Prior to the meetings, they were given the opportunity to speak with SP and were also emailed slides that provided background information about the project and the process of qualitative research more broadly. The materials explained that their role in the meetings was to consider the data from their perspective as young people. This was also emphasized within the meetings, and they were actively encouraged to give their thoughts and opinions throughout.

## Findings

**Figure 1.**
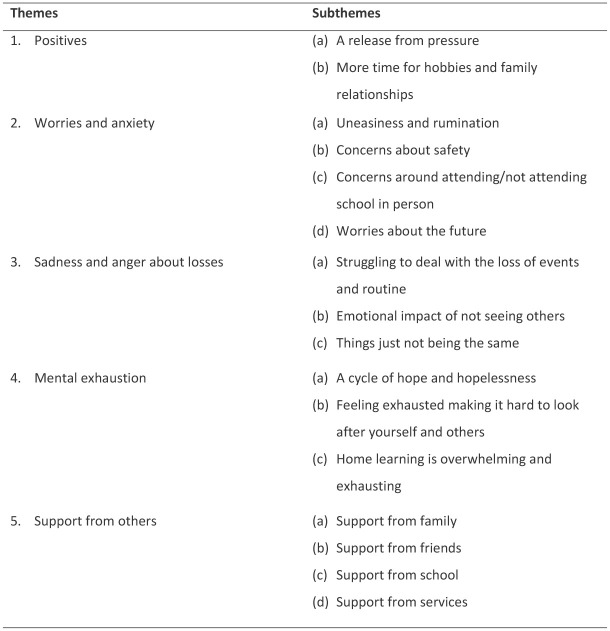
Themes and subthemes.

The current analysis focused on young people’s experiences across the pandemic, with a particular focus on their mental health and wellbeing, and how they coped. Their experiences were developed into five themes: (1) positives; (2) worries and anxiety; (3) sadness and anger about losses; (4) mental exhaustion; and (5) support from others. The themes and subthemes are presented in Figure 1. All names are pseudonyms.

### Theme 1: Positives

#### A Release From Pressure

Positive experiences described by young people were often focused on the early part of the pandemic and related to the consequences of school closures, such as a release from the stress of exams. They described the first lockdown as “a break” (Bridget, 15) from the pressures of school. The early stages of lockdown also brought about increased independence and autonomy over how to use their time.

Just focusing on me and what I need to do. (Aarushi, aged 15)

I liked working at my own pace and I liked not having to speak to anybody. (Bridget, aged 14)

#### More Time for Hobbies and Family Relationships

Many of the young people pointed out that they had a “lot more time free to like do [their] own things” (Aarushi, 15) in the early part of the pandemic as school, clubs, and organized activities came to an abrupt halt. This was seen positively by those who described the first lockdown “like a really long holiday” (Jacob, aged 11), with many young people seeing it as an opportunity to do “something new that [they had] never [done]” (Ethan, aged 14), such as growing vegetables in the garden.

For some young people, they were able to use this time for their hobbies and interests and this gave them something else to focus on other than the pandemic, helping them to connect with friends, or making things feel more normal.

I did lots of online courses and kept myself occupied. (Darcy, aged 16)

. . .most helpful thing to me is being able to read and play computer games and play guitar. Just carry on with my hobbies which I was doing before lockdown. (Adam, aged 14)

Being able to use this time to focus on themselves without having to “accommodate to other people” (Bridget, aged 14) was particularly valued by those with pre-existing mental health or physical health difficulties. In addition, there was a sense that more time spent as a family could sometimes lead family members to feel better or become “closer” (Ciara, aged 14).

It was, like, pretty much at least four times a week that we would do something altogether. Like all five of us as sisters.. . .and those days where we didn’t do anything, those days were kind of, like, low. (Amina, aged 13)

In some instances, there were fewer arguments than normal, “because everyone is a bit less stressed because they’re not really working that much” (Bridget, aged 14).

### Theme 2: Worries and Anxiety

#### Uneasiness and Rumination

Nevertheless, young people described feeling uneasy about the constant changes that were happening throughout the pandemic, such as rules around being able to see other people, schools closing and re-opening, and exams being canceled. They described feeling a sense of inner conflict; they either felt confused about the situation and government response or could understand different perspectives and felt conflicted about which to follow. Young people described feeling unsure whether seeing friends or taking part in activities was “the right thing” (Adam, aged 14) to do, given the risks of catching COVID.

It was mixed feelings because yes, it was nice socializing. But then it’s kind of selfish, isn’t it? (Aarushi, aged 15)

Ruminating and worrying about events that had already occurred that day, or during increased periods of free time was common, with young people describing “sitting around on [their] own getting stressed about work and things like that” (Fred, aged 15). For young people without a history of mental health difficulties and/or SEN/ND, the experience of “not knowing what’s happening with, like all sorts of things” (Adam, aged 14) was described in ways that implied that the level of stress was manageable. For example:

Er, a bit stressed like it kind of it wasn’t, like I didn’t feel like really stressed, but I mean it was kind of, on my mind and still is like a bit. . . (Adam, aged 14)

However, those with pre-existing mental health difficulties and/or SEN/ND described these changes having a serious impact on feelings of anxiety:

Not knowing when this lockdown was gonna end was horrible. Like even now when they you know say ‘oh, there’s a new tier, or there’s a new added bit to lockdown and now you can only meet this number of people’ like that still gives me panic attacks. . . (Harriet, aged 15)

#### Concern About Safety

Young people described remaining concerned about their own and other’s safety as restrictions lifted. Two young people from the north of England (where infection rates tended to be relatively high compared to the rest of the UK) who also had SEN/ND reported that, despite the measures in place, “it does feel unsafe” (Harriet, aged 15), especially in crowded areas where they would “end up getting quite stressed” (Fred, aged 15). Some young people “became more worried for my grandparents than for me” (Bethan, aged 15) and felt a responsibility for keeping others safe.

I’m thinking about something goes wrong and it’s my fault that people in my house end up with this virus. So I was quite stressed out about that. (Fred, aged 15)

#### Concerns Around Attending or Not Attending School in Person

With the closures of school to most young people extending from March to September 2020, there were mixed feelings about the return to school. Some young people described how much they missed the school environment and, as a result, felt “scared that [they] won’t be able to go to school for a long time” (Georgina, aged 11).

However, there was a recognition of the challenges of the school environment and young people, especially those who had pre-existing mental health difficulties or had experienced abuse or bullying at school, had often legitimate concerns about the return to school. Bridget reported that she “really did not want to” go back to school and was very anxious about returning. This related to:

the workload and having to see people when you haven’t seen people for so long. (Bridget, aged 14).

Jiro (aged 14) had experienced COVID-related racist abuse early in the pandemic before the first lockdown and described how he “didn’t want to be foreign,” and was very worried about going back to school and the abuse reoccurring.

At home its harder to learn, but it’s not as much stress from other people. (Jiro, aged 14)

Some were anxious or stressed about catching COVID in the school environment and potentially inadvertently infecting others:

I got a bit like paranoid about like touching stuff . . . so it kind of, at the same time as being really good, it was quite stressful. (Adam, aged 14)

As young people returned to school at the start of the new academic year, many returned to a changed environment from the one they had left. For those who had remained at school throughout lockdown, the once quiet environment became “hectic” and “loud” (Fiona, aged 15) as other students began to return. For others, the restrictions that had been introduced (e.g., mask wearing and restricted movement around buildings) were described as “all very confusing” (Jacob, aged 11) by young people.

#### Worries About the Future

Many young people reported feeling worried about their future. Some reported feeling worried “that it stays like this. . .not seeing people and having a normal life” (Darcy, aged 16).

Worries about the impact on their education were common across the age range but it was noticeable that older individuals had concerns around “how my future is going to be” (Fiona, aged 15) and preparation for GCSE exams, taken at the end of compulsory education.

I just wish that I could be able to know now. . . I feel confused by not knowing what like I’m prepared, I’m preparing for. (Adam, aged 14)

I mean, it is quite stressful to know how many exams I’ve got to get through, left when I’ve missed like 3, 4 months of school. (Bethan, aged 16)

Whereas for younger children, worries about the future appeared to be more broadly about not attending school.

I won’t be able to go to school for a long time. But apart from that, not really’ (Georgina, aged 11).

### Theme 3: Sadness and Anger About Losses

#### Struggling to Deal With the Loss of Events and Routine

Young people experienced multiple losses in their lives throughout the pandemic, from everyday routines and activities to significant life events (e.g., end-of-school-year events, events that related to their hobbies and interests, and exams), particularly in times of full lockdown. The loss of routine and events often left them feeling “bored, so I get upset. . .days just seem very long” (Naomi, aged 13), with some reporting that they “kind of spiral into depression because you don’t know what to do with yourself” (Fiona, aged 15). Not being able to go outside very much or exercise as part of hobbies made it “harder to get to sleep at night” (Darcy, 16) and “really, really sucked” (Harriet, aged 15).

While there were positives for some in spending more time with family, young people also found this hard, feeling trapped with no escape or outlet for built-up frustrations which led to an increase in emotional distress and irritability. Families “argued quite a bit more than usual” (Fred, aged 15) and young people also reported that they had a shorter temper than usual in lockdown.

I mean everything I could possibly be angry with, I was kind of angry with at some point. Erm. My parents, the government, coronavirus in general, yeah. (Adam, aged 14)

#### Emotional Impact of Not Seeing Others

One important loss for young people was not being able to spend time with friends and family out of the house. Young people felt they had “lost touch” (Adam, aged 14) with school and friends, causing them to feel socially isolated and described feeling “panic” (Ciara, aged 14) about this. The isolation was particularly difficult for those who “don’t have a phone that can text or anything” (Jacob, aged 11). Many felt that “it’s really sucked not being able to see [family]” (Harriet, aged 15) beyond those in the household, such as cousins and grandparents; causing them to feel “sad” or “depressed.” One of the interviewees described feeling “very, very upset” (Georgina, aged 11) about not being able to see her divorced parent.

The loss of face-to-face school was particularly isolating for many young people. They described it being “just stressful ‘cause . . .teachers find it harder to explain things over the internet to us” (Darcy, aged 16) or that there “wasn’t a lot of interaction” (Bethan, aged 15) when they wanted to ask a question. However, even when back at school with restrictions, there were elements of this that young people described as “horrible” because they had been placed in bubbles and could not mix these to spend time with their friends.

#### Things Just Not Being the Same

Staying in touch with friends via technology during lockdown and the return to hobbies, school, and other events were ultimately described as “frustrating” (Adam, aged 14). For example, although they appreciated technology allowing them to see and interact with people, they also explained that “it’s not really the same” (Bridget, aged 14), which they found “really, really sad and really upsetting” (Harriet, aged 15). It also felt much harder to communicate remotely than in person, finding texting “tedious ‘cause you can’t, you’ve got to try and, like, put everything into words and then you’ve got to try and like, it never seems to flow as well” (Ciara, aged 14).

When hobbies and outdoors social interaction were allowed, young people described it being not quite the same as it had been before.

Having to social distance . . .and like not being able to do half the stuff that you would be able to is sad. (Harriet, aged 15)

### Theme 4: Mental Exhaustion

#### A Cycle of Hope and Hopelessness

Young people described feeling caught in a loop of initially feeling optimistic, but as time went on, they felt fed up and lost hope:

. . .during the start [of lockdown], I think most of us kind of thought ‘oh it’s, it’s going to, kind of, be almost a holiday,’ but didn’t end up being like that. . . (Isabella, aged 12)

Along the way, events, such as the roll-out of the vaccination program, made some feel “a lot more hopeful” (Harriet, aged 15), and restrictions being lifted made others feel “happy when everything opened up a bit, because, like, there was hope. . .” (Ciara, aged 14). However, repeated lockdowns and restrictions caused young people to lose that hope, worry, and to have to prepare themselves for things not returning to normal:

It’s never really going to end. We’re always gonna have to be more careful than we were before. (Isabella, aged 12)

#### Feeling Exhausted Making It Hard to Look After Yourself and Others

Young people reported that they felt tired and exhausted throughout the pandemic.

. . .it was really wearing, and it was extremely tiring at the start of the pandemic ‘cause I just didn’t have enough energy to, like, do anything, ‘cause, like, my brain was just scrambled. (Harriet, aged 15)

Young people commonly found each of the two national lockdowns more and more “tiring” (Adam, aged 14). They felt too tired to keep in touch with friends, which seemed to take more energy than it did before; finding that they “didn’t message anyone back for, like, a few months because it was just too much to face” (Harriet, aged 15). Balancing academic work and other demands could be difficult, particularly for those with extra responsibilities at home.

I’m trying to attend every live lesson. . .and mum is disabled and struggles to get around. . . now dad is at work, it’s more, I’m helping out mum. . .I’m having a big dip in energy (Jacob, aged 11)

#### Home Learning is Overwhelming and Exhausting

Many found the move to home learning during the first lockdown difficult. Young people themselves found it stressful trying to do schoolwork in their home environment, which had not been designed as an environment for learning and where they often lacked resources and space. The requirement to organize their own work without the support of teachers was “a bit of a shock at first” (Adam, aged 14), which made it hard to then work effectively.

I felt like it was, it was quite hard to get the work done. I’ve got a lot of distractions around me. (Ethan, aged 14)

My school didn’t have online lessons, they kind of sent work home, but it was, I couldn’t get it done, like motivating yourself to get like through a whole pile of papers was not really too fun so I didn’t really get any work done for like 6 months. (Harriet, aged 15)

During the second national lockdown, the workload felt heavier for some:

I literally just get upset for no reason. Well not for no reason, because the teachers always set so much work. And I just feel really overwhelmed. (Amina, aged 13)

In addition, the increased effort needed to maintain concentration for teaching when delivered online was overwhelming and described as “a lot more tiring because it’s so easy to just drift off an online lesson” (Adam, aged 14).

Once lockdowns were lifted and schools re-opened, there was further stress for those who were unable to attend school in person due to the requirement to self-isolate:

It cause disruptions because I was sent home, in the September to December time, I was sent home twice and each time it was for two weeks. (Aarushi, aged 15)

### Theme 5: Support From Others

#### Support From Family

Young people were generally positive about the support they received from their families in relation to understanding and coping with COVID. They felt that it was “better hearing [COVID information] from [mum] because she’s more reassuring” (Georgina, aged 11). Many young people felt grateful for being able to talk to their parent or carer about how they were feeling and coping.

I can be open with my mum, but not many other people (Fred, aged 15).

Some also found the presence of their siblings “helps immensely” to cope through lockdown, speculating that their absence would have caused them to “feel alone” (Aarushi, aged 15).

Young people also felt conflicted between feeling “concerned for everyone” (Ciara, aged 14) in their family but also wanting or needed support from their family.

I mean, I know it’s difficult when they’re feeling stressed too. But when they can, I mean, I always get support if I’m feeling bad about something. (Adam, aged 14)

Nevertheless, for some young people, there were limits to the support parents or carers could provide.

We just don’t really click on that level with mental health, which is no problem ‘cause, you know, not everyone is going to understand. (Harriet, aged 15)

#### Support From Friends

For most young people, being able to talk to friends and keep in contact was described as a “lifesaver” (Harriet, aged 15). They found that talking to friends about their emotions and about how to deal with the pandemic restrictions was “something that helped me cope” (Naomi, aged 13):

I talk about different things with my friends than I would with my parents. It is being able to discuss stuff with my friends that’s, it’s a good way to, like, stay mentally calm. (Bethan, aged 15)

However, there were some young people who reported “not really” (Georgina, aged 11) talking to their friends much about how they were feeling about the pandemic and that talking to friends “would[n’t] have made a difference” (Ethan, 14) or that their friends wouldn’t “be of use” (Fred, aged 15).

I talk to my friends but they’re the same age as me so they can’t really do much to help me. (Naomi, aged 13)

For those attending school through lockdown, a major benefit of this was being able to spend time with and gain support from their friends in person. Once schools reopened after periods of lockdown, some young people described feeling “happy because you got to, like, see everyone again” (Claire, aged 12).

#### Support From School

Young people’s feelings about the support they received from school were often mixed. Whilst some felt grateful for the educational or mental health support that school provided, others felt that school could have done more to support them, especially in the first lockdown. Some felt abandoned by school in relation to support around the young person’s wellbeing and the provision of resources to be able to learn at home.

They didn’t really care what was properly going on. . .[they could have]. . .Given me a laptop to use. . .they could’ve phoned up more and said, what’s happening. (Ethan, aged 14)

Most felt that by the second national lockdown, this support had improved. This appeared to relate to greater opportunity for regular interactions with teachers through online lessons or, for some, because they were able to attend school in person.

I think ‘cause I’m going into school now, they they definitely make sure I’m OK. (Harriet, aged 15)

#### Support From Services

For those who required professional support, accessing mental health services was helpful, even if they were initially reluctant. However, as services moved to online provision, especially early in the pandemic, this was not always well received.

It was really helpful and now I’m doing a lot better and I can still see her now in person ‘cause it’s a medical appointment. So that’s really really helpful and important like I would have struggled if that went online. (Harriet, aged 15)

When we did have a meeting it would be online, making it difficult, even more difficult than it normally is, to explain how I feel in real life when I’m there (Fiona, aged 15)

. . . I didn’t really feel it was, I don’t know, confidential. . .just doesn’t feel like I can be as open. (Fred, aged 15)

## Discussion

The present study sought to qualitatively explore the experiences of 17 young people, aged 11 to 16 years, in relation to their mental health and wellbeing and how they coped during the COVID-19 pandemic within the UK. Through thematic analysis of young people’s interviews, we developed five themes; (1) positives (especially during the first lockdown), such as a release from social and educational pressures, as well as more time to spend on things they enjoyed and with family; (2) worries and anxiety, including feelings of uneasiness about doing the right thing, safety, attending (or not attending) school in person, and worries about the future, (3) sadness and anger about losses, including the loss of events and routines, not seeing others, and things not being the same; (4) mental exhaustion, due to a continuous cycle of hope and hopelessness, and home learning feeling overwhelming and exhausting; and (5) support from family, friends, school, and services. Within each theme, young people identified factors that related to themselves, their family, and the wider environment that had both positive and negative effects on their wellbeing and ability to cope.

The young people in this study identified a range of factors that appeared to facilitate resilience over the course of the pandemic, relating to them as an individual, their family, the environment, and school/support services (Luthar & Cicchetti, 2000). During the first national lockdown in particular, some young people appeared to enjoy the opportunity for greater independence and autonomy—an important normative process during this developmental stage (Steinberg & Silverberg, 1986). For those young people who were able to be self-directed in their learning, initiate activities and have access to resources that facilitated interests (e.g., a garden), this was seen positively. Young people also identified other factors that could be seen as a “developmental asset” (Roehlkepartain & Blyth, 2020), such as having close, supportive relationships with parents and siblings who they enjoyed spending time with and were a source of support. Given the critical importance of peers during adolescence (Blakemore & Choudhury, 2006; Brown & Klute, 2006), it was important for young people that they could continue to maintain these relationships remotely, such as through video-calling, texting, and social media; they were seen as a “lifesaver” in terms of helping them cope and “stay mentally calm.” Finally, support from school and mental health services was also important for those who needed it.

Nevertheless, at this point in the pandemic, young people in this study described struggling emotionally, with feelings of worry/anxiety, sadness, and anger. This is consistent with previous findings from both quantitative and qualitative research (Burgess et al., 2022; Children’s Parliament, 2020; Creswell et al., 2021; McKinlay et al., 2022; NHS Digital, 2020; O’Sullivan et al., 2021; Pellicano et al., 2022; Scott et al., 2021). For example, several quantitative studies identified a decline in wellbeing and increase in emotional, behavioral, and concentration/hyperactivity difficulties related to the pandemic and its associated restrictions (Children’s Parliament, 2020; Creswell et al., 2021; NHS Digital, 2020). Increases in stress and anxiety, as well as difficulties coping with the experiences of losses, were commonly reported across many of the previous qualitative studies (Burgess et al., 2022; McKinlay et al., 2022; O’Sullivan et al., 2021; Pellicano et al., 2022; Scott et al., 2021). In our study, a key source of emotional distress appeared to be feeling isolated and trapped at home and not being able to spend time with friends. Given that adolescence involves an increased need for social connection, peer acceptance, and belonging (Collins, 1997), it is likely that being separated from peers was especially challenging. In addition, racism featured in both our study and that of Burgess et al. (2022), where participants from ethnic minorities were held responsible and on the receiving end of abuse for COVID-19, causing a great deal of distress. This must be seen in the context of system racism and discrimination as well as other significant stressors, such as greater social deprivation and higher mortality rates from Covid-19 for those from ethnic minority backgrounds compared to their White counterparts (Morales & Ali, 2021; Smith et al., 2020).

In our study, young people’s background characteristics appeared to play a role in their experiences, consistent with previous quantitative literature. The young people in the sample with pre-existing mental health difficulties, special educational needs, and neurodevelopmental disorders appeared to find uncertainty and change, such as an increase in restrictions or the return to school after lockdown, particularly challenging. In some cases, they made direct links between recalling events related to the pandemic, such as increased restrictions, and emotional distress, such as experiencing a panic attack. This is consistent with survey data that have shown elevated patterns of mental health difficulties during the pandemic in young people with special educational needs and neurodevelopmental disorders (Creswell et al., 2021; Raw et al., 2021). Scott et al. (2021) also reported that participants in their sample with pre-existing mental health difficulties found that these were exacerbated during periods of lockdown or increased restrictions. Although in our study, young people’s worries about the impact on their education were common across the age range, it was noticeable that older individuals had specific concerns about exams and their future. This was consistent with McKinlay et al. (2022), which included participants up to 24 years old who described feeling uncertain about their education and were worried about not meeting educational expectations. It was evident that young people who lacked resources, such as access to a laptop for schoolwork or (quiet) space found this created additional stress.

It was notable that our findings around young people feeling mentally exhausted and experiencing a cycle of hope and hopelessness have not been identified in previous studies. Young people described difficulty trying to make decisions around their own wants and needs in relation to socializing, whilst being aware this could have repercussions for the safety of others. Given that adolescence involves an increased need for social connection, peer acceptance, and belonging (Collins, 1997), and greater susceptibility to peer influence (Tomova et al., 2021), it is perhaps unsurprising that managing social contact, for example, deciding whether to see friends and maintain social distancing, was a particular challenge for this age group. The longer time period for the current study compared to others meant that we captured a cyclical pattern of emotions, as young people went from feeling hopeful, back to hopeless, as positive events (e.g., case numbers falling, leading to the easing of restrictions) were then undercut by new negative events (e.g., a new variant emerging, leading to increases in cases and a tightening of restrictions and a further lockdown). It may have been difficult for young people to have a sense of self-efficacy/personal power or a positive view of their personal future, factors that have been shown to be associated with positive coping (Scales et al., 2000).

Our findings suggested that during this period, young people found support from multiple sources, including parents/carers, siblings, friends, school, and mental health services, but that this was not always sufficient. Young people varied in who they sought support from, and in their ability to access support (and other people’s ability to successfully provide support). Our finding that there were gaps in the provision of support from schools, especially early in the pandemic, and that the online support provided by mental health services could be problematic for some young people replicates findings from other studies; for example, McKinlay et al. (2022) identified that there was a lack of support from schools around young people’s wellbeing, and Pellicano et al. (2022) reported that autistic young people found online support from health services to be a negative experience. We also found that young people’s ability to seek support could also vary; for example, being exhausted could get in the way of being able to reach out to friends, and it could be hard to ask a stressed parent or carer for support.

### Implications

Clearly, the picture is still developing in terms of the consequences of the pandemic for young people. While the purpose of this qualitative study was to provide an in-depth understanding of the experiences of the young people rather than generalizable findings, there is a great deal of consistency with the broader literature. Thus, there are several potential implications. It is important that parents/carers and schools recognize the emotional impact the pandemic and associated restrictions had on young people and look for ways to minimize this going forward. Academic pressure and worries around workloads were common and so initiatives to address gaps in learning will need to be sensitive to these concerns without creating additional stress. To ensure equity of access to education if there are future episodes of home-schooling, it will be important that there is a reliable technological infrastructure available for successful blended/online learning (Starkey et al., 2021) and that research examines how this delivery method can be optimized. Given the cycles of hopelessness and feelings of sadness and anger, prioritizing social activities and hobbies and interests that give young people a sense of identity, purpose, and enjoyment will also be important. Given the ongoing nature of the COVID-19 pandemic and the high levels of unease and anxiety reported in the study, young people are also likely to benefit from help in learning how to manage and live with uncertainty. It will be crucial to continue to track the impact of the pandemic on young people’s emotional wellbeing. Parents, carers, and professionals supporting young people should be on the lookout for young people who may be vulnerable, such as those with mental health difficulties, special educational needs, neurodevelopmental difficulties, and additional caring responsibilities, to be able to monitor them and provide support as needed. Given the low rates of identification of mental health difficulties in young people, it may be necessary to develop school-based programs to identify young people who are experiencing difficulties at a level that cause distress and interference and would benefit from support. Finally, the findings demonstrate the importance of providing support to young people in multiple ways and addressing barriers for young people to seeking support, such as normalizing of mental health difficulties, and finding opportunities for regular, informal conversations about mental health rather than relying on young people to initiate help-seeking (Radez et al., 2021). Accelerating the provision of evidence-based support and interventions to those who require it will be important in helping young people to bounce back from the impact of the pandemic as we move forward.

### Strengths and Limitations

This study benefits from several strengths; interviews explored experiences including two periods of national lockdowns/tightened restrictions, and purposive sampling was used to recruit a diverse sample of adolescents from a variety of locations and backgrounds across the UK. The study was conducted with a high level of rigor and credibility and benefited from the inclusion of young people as members of the research team who were involved in the analysis and write-up. However, there are limitations to this study that should be considered. We were not able to capture experiences of young people from groups who were not represented in the study (e.g., young people from Northern Ireland). We did not obtain information about some characteristics (e.g., whether young people identified as being LGBTQ+). Further research is needed to gain a greater understanding of the experiences of young people from these and other groups. We did not find gender to be associated with experiences but did not explicitly ask about this, and therefore, the lack of findings may reflect what was covered in the interviews. While we sought to gain a broad perspective on young peoples’ experiences at a critical time point, there may be advantages to recruiting more homogenous samples to provide more in-depth and contextualized findings, particularly where there were relatively few young people in the sample with a particular characteristic. Despite the exploration of experiences across the multiple lockdowns being an advantage of this study, it also meant a reliance on memory of experiences from up to 1 year prior. Two of the young people chose to have a parent/carer present for the interview, and four of the young people were interviewed in an area where other family members were present in the background; although these young people talked openly about their experiences, inevitably the presence of family members is likely to have influenced what was said. Finally, participants were interviewed between December 2020 (when the second national lockdown was expected but not yet announced) and February 2021 (when the second national lockdown was approaching an end), within which times the current pandemic situation was very different. Although participants interviewed earlier did not appear to provide more positive views than those interviewed later, the timings of the interviews may have impacted the way participants described their experiences. As we approach differences phases of the pandemic, it will be important to continue to capture the voices of young people through further research.

## Conclusion

To conclude, this study sheds an important light on the experiences of young people’s emotional wellbeing, mental health, and coping during a global pandemic. Consistent with other studies, we found that young people experienced some positives early in the pandemic but overall, feelings of worry and anxiety, sadness, and anger about losses were common. Notably, we also found that by this point of the pandemic, young people felt mentally exhausted due to a continuous cycle of hope and hopelessness and that young people’s experiences appeared to vary according to key background characteristics. Young people received support in multiple ways but there could be barriers to successfully accessing support, such as feeling too exhausted to reach out to friends, or concerns about receiving online support from services. Moving forward, it will be crucial for those supporting young people to recognize the emotional impact of the pandemic on young people, provide opportunities for them to reconnect with others and engage in hobbies and interests, and help them to manage the uncertainty as we move to new phases of the pandemic. Continued measurement of young people’s emotional wellbeing and mental health, initiatives to identify young people who have been struggling and the provision of support (including evidence-based and accessible interventions) will be important for protecting young people from future adversities as we emerge from the pandemic.

## Research Data

sj-docx-1-jar-10.1177_07435584231151902 – for How the COVID-19 Pandemic Affected Young People’s Mental Health and Wellbeing in the UK: A Qualitative Study sj-docx-1-jar-10.1177_07435584231151902 for How the COVID-19 Pandemic Affected Young People’s Mental Health and Wellbeing in the UK: A Qualitative Study by Samantha Pearcey, Lowrie Burgess, Adrienne Shum, Eshal Sajid, Milly Sargent, Marie-Louise Klampe, Peter J. Lawrence and Polly Waite in Journal of Adolescent Research

## References

[bibr1-07435584231151902] Blakemore S. J. Choudhury S. (2006). Development of the adolescent brain: Implications for executive function and social cognition. Journal of Child Psychology and Psychiatry, 47(3–4), 296–312. 10.1111/j.1469-7610.2006.01611.x 16492261

[bibr2-07435584231151902] Braun V. Clarke V. (2006). Using thematic analysis in psychology. Qualitative Research in Psychology, 3(2), 77–101.

[bibr3-07435584231151902] Braun V. Clarke V. (2013). Successful qualitative research: A practical guide for beginners. SAGE.

[bibr4-07435584231151902] Braun V. Clarke V. (2021). To saturate or not to saturate? Questioning data saturation as a useful concept for thematic analysis and sample-size rationales. Qualitative Research in Sport Exercise and Health, 13, 201–216.

[bibr5-07435584231151902] Brown B. B. Klute C. (2006). Friendships, cliques, and crowds. In Adams G. R. Berzonsky M. D. (Eds.), Blackwell handbook of adolescence (pp. 330–348). Blackwell Publishing Ltd.

[bibr6-07435584231151902] Burgess R. A. Kanu N. Matthews T. Mukotekwa O. Smith-Gul A. Yusuf I. Lamptey I. McCauley N. Wilson R. Pirisola M. Gul M. (2022). Exploring experiences and impact of the COVID-19 pandemic on young racially minoritised people in the United Kingdom: A qualitative study. PLoS One, 17, e0266504. 10.1371/journal.pone.0266504 PMC9067664 35507595

[bibr7-07435584231151902] Children’s Parliament. (2020). How are you doing? A report on the findings from the how are you doing? Survey using data from April, May and June 2020. https://www.childrensparliament.org.uk/wp-content/uploads/HOW-ARE-YOU-DOING-SURVEY-REPORT-August-2020.pdf

[bibr8-07435584231151902] Collins W. A. (1997). Relationships and development during adolescence: Interpersonal adaptation to individual change. Personal Relationships, 4(1), 1–14. 10.1111/j.1475-6811.1997.tb00126.x

[bibr9-07435584231151902] Creswell C. Shum A. Pearcey S. Skripkauskaite S. Patalay P. Waite P. (2021). Young people’s mental health during the COVID-19 pandemic. The Lancet Child & Adolescent Health, 5(8), 535–537. 10.1016/S2352-4642(21)00177-2 34174991 PMC9765398

[bibr10-07435584231151902] Goossens L. Marcoen A. (1999). Relationships during adolescence: Constructive vs. negative themes and relational dissatisfaction. Journal of Adolescence, 22(1), 65–79. 10066332 10.1006/jado.1998.0201

[bibr11-07435584231151902] Luthar S. S. Cicchetti D. (2000). The construct of resilience: Implications for interventions and social policies. Development and Psychopathology, 12(4), 857–885. 11202047 10.1017/s0954579400004156 PMC1903337

[bibr12-07435584231151902] Luthar S. S. Cicchetti D. Becker B. (2000). The construct of resilience: A critical evaluation and guidelines for future work. Child Development, 71(3), 543–562. 10953923 10.1111/1467-8624.00164 PMC1885202

[bibr13-07435584231151902] Malterud K. Siersma V. D. Guassora A. D. (2016). Sample size in qualitative interview studies: Guided by information power. Qualitative Health Research, 26(13), 1753–1760. 26613970 10.1177/1049732315617444

[bibr14-07435584231151902] McKinlay A. R. May T. Dawes J. Fancourt D. Burton A . (2022). ‘You’re just there, alone in your room with your thoughts’: A qualitative study about the psychosocial impact of the COVID-19 pandemic among young people living in the UK. BMJ Open, 12(2), e053676. 10.1136/bmjopen-2021-053676 PMC8829834 35140155

[bibr15-07435584231151902] Morales D. R. Ali S. N. (2021). COVID-19 and disparities affecting ethnic minorities. Lancet, 397(10286), 1684–1685. 33939952 10.1016/S0140-6736(21)00949-1 PMC9755653

[bibr16-07435584231151902] NHS Digital. (2020). Mental health of children and young people in England, 2020: Wave 1 follow up to the 2017 survey. https://digital.nhs.uk/data-and-information/publications/statistical/mental-health-of-children-and-young-people-in-england/2020-wave-1-follow-up/copyright

[bibr17-07435584231151902] NIHR. (2020). Centre for engagement and dissemination - Recognition payments for public contributors. https://www.nihr.ac.uk/documents/centre-for-engagement-and-dissemination-recognition-payments-for-public-contributors/24979

[bibr18-07435584231151902] O’Sullivan K. Clark S. McGrane A. Rock N. Burke L. Boyle N. Joksimovic N. Marshall K. (2021). A qualitative study of child and adolescent mental health during the COVID-19 pandemic in Ireland. International Journal of Environmental Research and Public Health, 18(3), 1062. 10.3390/ijerph18031062 33504101 PMC7908364

[bibr19-07435584231151902] Patton M. (2014). Qualitative research & evaluation methods: Integrating theory and practice. SAGE publications. Inc.

[bibr20-07435584231151902] Pellicano E. Brett S. Den Houting J. Heyworth M. Magiati I. Steward R. Urbanowicz A. Stears M. (2022). COVID-19, social isolation and the mental health of autistic people and their families: A qualitative study. Autism, 26(4), 914–927. 10.1177/13623613211035936 34362263

[bibr21-07435584231151902] Radez J. Reardon T. Creswell C. Lawrence P. J. Evdoka-Burton G. Waite P. (2021). Why do children and adolescents (not) seek and access professional help for their mental health problems? A systematic review of quantitative and qualitative studies. European Child & Adolescent Psychiatry, 30(2), 183–211. 10.1007/s00787-019-01469-4 31965309 PMC7932953

[bibr22-07435584231151902] Raw J. A. L. Waite P. Pearcey S. Shum A. Patalay P. Creswell C. (2021). Examining changes in parent-reported child and adolescent mental health throughout the UK’s first COVID-19 national lockdown. Journal of Child Psychology and Psychiatry, 62(12), 1391–1401. 10.1111/jcpp.13490 34327726 PMC8447308

[bibr23-07435584231151902] Roehlkepartain E. C. Blyth D. A. (2020). Developmental assets. In Hupp S. Jewell J. D. (Eds.), The encyclopedia of child and adolescent development (pp. 1–13). Wiley-Blackwell.

[bibr24-07435584231151902] Scales P. C. Benson P. L. Leffert N. Blyth D. A. (2000). Contribution of developmental assets to the prediction of thriving among adolescents. Applied Developmental Science, 4(1), 27–46.

[bibr25-07435584231151902] Scott S. McGowan V. J. Visram S. (2021). ‘I’m gonna tell you about how Mrs Rona has affected me’. Exploring young people’s experiences of the COVID-19 pandemic in North East England: A qualitative diary-based study. International Journal of Environmental Research and Public Health, 18(7), 3837. 10.3390/ijerph18073837 33917557 PMC8038818

[bibr26-07435584231151902] Shulman S. (1993). Close relationships and coping behavior in adolescence. Journal of Adolescence, 16(3), 267–283. 8282898 10.1006/jado.1993.1025

[bibr27-07435584231151902] Smith K. Bhui K. Cipriani A. (2020). COVID-19, mental health and ethnic minorities. Evidence-Based Mental Health, 23, 89–90. 32680834 10.1136/ebmental-2020-300174 PMC7418618

[bibr28-07435584231151902] Starkey L. Shonfeld M. Prestridge S. Cervera M. G. (2021). Covid-19 and the role of technology and pedagogy on school education during a pandemic. Technology Pedagogy and Education, 30(1), 1–5.

[bibr29-07435584231151902] Steinberg L. Silverberg S. B. (1986). The vicissitudes of autonomy in early adolescence. Child Development, 57, 841–851. 3757604 10.1111/j.1467-8624.1986.tb00250.x

[bibr30-07435584231151902] Tomova L. Andrews J. L. Blakemore S.-J. (2021). The importance of belonging and the avoidance of social risk taking in adolescence. Developmental Review, 61, 100981. 10.1016/j.dr.2021.100981

[bibr31-07435584231151902] Weist M. D. Freedman A. H. Paskewitz D. A. Proescher E. J. Flaherty L. T. (1995). Urban youth under stress: Empirical identification of protective factors. Journal of Youth and Adolescence, 24(6), 705–721.

[bibr32-07435584231151902] Widnall E. Winstone L. Mars B. Haworth C. Kidger J. (2020). Young people’s mental health during the COVID-19 pandemic: Initial findings from a secondary school survey study in South West England. https://sphr.nihr.ac.uk/wp-content/uploads/2020/08/Young-Peoples-Mental-Health-during-the-COVID-19-Pandemic-Report-Final.pdf

