# Supplementary material for: How the COVID-19 Pandemic Affected Young People’s Mental Health and Wellbeing in the UK: A Qualitative Study
Source: J Adolesc Res. 2023 Feb 2;39(6):1573–600. doi: 10.1177/07435584231151902 (PMC9899677; doi:10.1177/07435584231151902)
Supplement: sj-docx-1-jar-10.1177_07435584231151902 – for How the COVID-19 Pandemic Affected Young People’s Mental Health and Wellbeing in the UK: A Qualitative Study [file sj-docx-1-jar-10.1177_07435584231151902.docx]

**How the COVID-19 pandemic affected young people’s mental health and wellbeing in the UK: A qualitative study**

**Electronic Supplementary Materials**

**Indicative Topic Guide**

- How have the last few months during the pandemic been for you?
- How have things changed for you and your family?
  - Prompt for family/peer relationships, wellbeing/health, education/school, activities
  - What things have you enjoyed/appreciated/found easier?
  - What have you found difficult/challenging?
- How were you affected by lockdown and schools closing in March?
  - What did you think, how did you feel?
  - How did this affect interactions with your family?
  - What changes did you experience in family dynamics, emotions, arguments etc…?
  - How did you keep in contact with friends/family over lockdown? Did this influence how you felt about being at home?
  - Any changes over time in how you felt about it?
- How did you feel about some children returning to school in June?
- When were you first able to meet up with someone outside of your family/home bubble?
  - How did you feel?
  - Did seeing/meeting/contacting others support you?
- Have you returned to school?
  - How did you feel about the return to school?
  - What do you think about the return to school?
- How did you feel about the second lockdown?
  - Have things changed at home? School?
  - Did you keep in touch with friends?
  - What are your fears?
  - What are your hopes?
  - Can you see positives in this second lockdown?
  - How do your emotions differ compared to the first lockdown?
- Are there things have helped you cope through this time?
  - What things? From where?
  - Are there things that you have felt you have needed support for?
  - What support have you/would you have found helpful? From where?
- What things have been unhelpful? Where have these come from?
- Looking to the future, are there things over the next few months that you think might make life easier or more difficult?
  - If difficult, what would help you cope?
- If we were in a similar situation again in the future, what would you like to be done differently- either by yourself or other members of your family, or people and organisations around you?
- What would you tell another young person going into this situation?
- Is there anything else that you wanted to talk about?
